# Development of a Model for Predicting the Thermophysical Properties of Carbon Materials and Proposal of Manufacturing Conditions Using the Model

**DOI:** 10.1002/ansa.70031

**Published:** 2025-07-30

**Authors:** Masayoshi Matsubara, Ryo Sasaki, Jun P. Takahara, Shinji Moritake, Yasuyuki Harada, Hiromasa Kaneko

**Affiliations:** ^1^ Department of Applied Chemistry School of Science and Technology Kanagawa Japan; ^2^ Mitsubishi Chemical Corporation Tokyo Japan

**Keywords:** genetic algorithm, genetic‐algorithm‐based process variables and dynamics selection, machine learning, needle coke, process conditions, time delay

## Abstract

A steelmaking method using electric furnaces is attracting attention in the iron and steel industry, and a carbon material called needle coke is used as an aggregate for the electrode in electric steelmaking. The performance of needle coke as an aggregate for electrodes in steelmaking is greatly affected by the quality of the needle coke, which depends on the ingredients of the raw materials and the process conditions. Because the raw material ingredients are not always constant and depend on the place and time they are produced, the quality of the needle coke is not stable under the same process conditions. Therefore, it is necessary to optimise the process conditions. In this study, to optimise the process conditions using machine learning, a model was constructed to predict the thermophysical properties of needle coke from the raw material ingredients and process conditions based on previous data. Because the subject plant is operated in a dynamic process and there is a time delay in the previous data, the genetic‐algorithm‐based process variables and dynamics selection method, which selects the time delays and process variable regionally, was studied. Furthermore, inverse analysis was performed on a sample whose quality was considered to be outside the specifications based on the previous data, with the aim of controlling the quality within the product specifications by changing only the process conditions.

## 1 Introduction

The steelmaking methods used in the steel industry include the blast furnace method and the electric furnace method. The electric furnace method has attracted attention from the viewpoint of environmental protection and recycling because of the reuse of steel scrap as the raw material and its higher energy consumption efficiency [1] and lower CO_2_ emissions [2] than the blast furnace method. Thus, the global needle coke market is continuously growing (from USD 3.54 billion in 2017 to USD 4.15 billion in 2021) [3]. In this method, a carbon material called needle coke [4, 5] is used as an aggregate for furnace electrode in the electric furnace for steelmaking. Needle coke has high thermal and electrical conductivity, high density, low thermal expansion coefficient, and resistivity [6, 7]. It is widely used in metallurgy, namely, in the steel and aluminium industries in the production [8, 9], in the production of lithium‐ion batteries [10, 11, 12], high‐power graphite electrodes and electrode materials for supercapacitors [13, 14, 15], in addition to as an aggregate for furnace electrodes. When needle coke is used as an aggregate for furnace electrodes in electric furnace steelmaking, its performance as an aggregate for furnace electrodes is affected by the quality of the needle coke [16]. To achieve high performance as an aggregate for furnace electrodes, it is necessary to produce needle coke with excellent quality, but it is difficult to control the needle coke to the target quality [17, 18, 19, 20].

The quality of needle coke is greatly affected by the raw materials and process conditions [21, 22, 23]. The components of the raw materials are represented by a mixture of several raw material ingredients, and the raw material ingredients vary depending on their origin and the year [6, 24]. Even when the needle coke is produced under constant process conditions, the quality of the needle coke may not be stable, and the product can be outside the specifications because the components of the raw materials are different [25, 26]. To maintain the quality of needle coke within the target range, it is necessary to appropriately vary the process conditions for each raw material. The process conditions are proposed based on experience, and trial and error are required to maintain the quality of the needle coke within the specifications. However, even when needle coke is produced under proposed process conditions based on experience, off‐specification products are sometimes produced. This is because only some variables can be considered. It is expensive to perform repetitive operations under proposed process conditions based on experience. Therefore, to propose appropriate process conditions for each raw material, it is necessary to comprehensively consider the relationship between the raw materials, process conditions, and quality of the product, needle coke.

In this study, we propose a method for modelling the above relationship using machine learning. The advantage of the proposed method is that it can simultaneously consider all of the variables, which are difficult for humans to empirically consider, and propose process conditions based on previous data without repeatedly running.

The objective variables (*Y*) are the thermophysical properties related to the needle coke. The explanatory variables (*X*) are the raw material characteristics, such as the properties and composition, and process conditions, such as the temperature and pressure. A machine learning model, *Y* = *f*(*X*), was constructed. By inputting the generated *X* values into this model, the predicted *Y* values can be output. This makes it possible to predict *Y* values without conducting operations on candidates of *X*.

The subject plant was operated in a dynamic process with uncertain time delays between *X* and *X*, and *X* and *Y*. Because the values of each variable in the dataset are recorded on a particular date, the time between the processes must be considered. Because there are uncertain time delays between the processes from the raw material to the product, it is necessary to capture the time delays. Thus, a method for selecting the process variables and time delays using a genetic algorithm (GA), the GA‐based process variables and dynamics selection (GAVDS) method [27], was used. This method can select process variables considering the time delays from the raw material to the product, and a machine learning model for predicting the product property is constructed between the selected variables and product properties. In the conventional method, process variables and time delays are selected. In the proposed method, process variables and time delays can be selected for *X* for which time delay needs to be considered, and process variables can be selected for *X* for which time delay does not need to be considered.

Finally, we propose the optimal process conditions using the model. A sample that was considered to be outside the specifications was analysed. One million samples were generated by fixing the raw material ingredients to the same values as the sample and changing only the values of the process conditions. The process conditions of the sample with the best results, in which the product was within the specifications, among the results of all the generated samples, were used as the optimal process conditions in this study. This method makes it possible to search for optimal process conditions even when only the raw material ingredients are given and the processes are not operated.

Although mechanism‐based kinetic modelling has been successfully applied to understand carbonisation and thermal decomposition processes under controlled laboratory conditions [28, 29, 30], such approaches are not feasible in this study. This is because the dataset used was obtained from a commercial‐scale dynamic process, in which the reaction mechanisms, kinetic parameters, and detailed feedstock compositions are not explicitly known. The raw materials consist of complex and variable mixtures depending on the origin and time of procurement, and the process operates under fluctuating conditions. Therefore, constructing a deterministic mechanistic model is impractical. This limitation emphasises the significance of our proposed data‐driven approach, which allows us to model and optimise process conditions directly from historical data, even in the absence of mechanistic information.

## 2 Methods

### 2.1 Dataset

**FIGURE 1:**
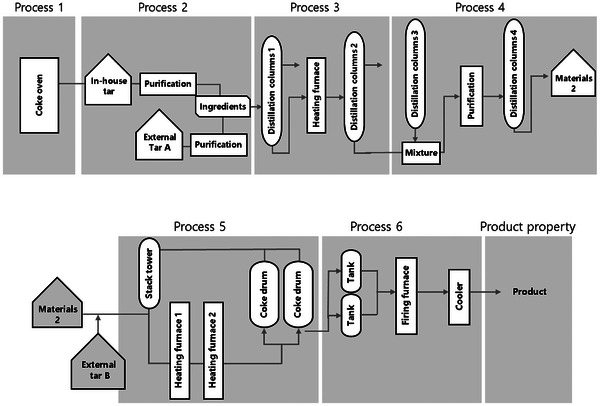
Flow of the processes.

We analysed the data obtained from the operation of a column at Mitsubishi Chemical Corporation from 2012 to 2022. The dataset consisted of the operation data from process 1 to process 6 as *X*, and two thermophysical properties of the needle coke as *Y* were used. The process flow from process 1 to process 6 is shown in Figure 1. In‐house tar is sourced from coke ovens, and needle coke from Mitsubishi Chemical Corporation is produced using tar derived from these ovens. We used 569 variables as *X*, including the raw material composition and process operating conditions. The process operating parameters were mainly parameters such as temperature, flow rate, pressure, and gas volume, while the raw material parameters were mainly parameters related to molecular weight, molecular structure, specific gravity, and impurities. The number of operating and material parameters for each process is listed in Table 1. Thermophysical properties A and B were used as *Y*.

**TABLE 1:** The number of operational parameters and raw material parameters for each process.

	Process 1	Process 2	Process 3	Process 4	Process 5	Process 6	External tar B
Operating parameters	43	0	61	123	100	80	0
Material parameters	27	16	55	27	19	0	18

Care must be taken when applying this as a time‐delayed dataset because the intermediate products from several lots of process 5 are mixed in process 6, as shown in Figure 1. Originally, several lots of process 5 were mixed before running process 6. In this study, the data of process 6 were copied, and each of the several lots of process 5 was considered to be one sample. For example, when there were three intermediate products that had gone through process 5, they were fed into process 6 after mixing the three intermediate products. In process 6, the data were from an operation using a mixture of the three intermediate products that went through process 5. To capture the time delays, the recorded data for process 6 was copied into three pieces of data, each sample corresponding to a lot of process 5, for a total of three samples of operation data. The value of *Y* was copied and linked in the same manner as for process 6. This method can consider the time delays between the samples, but the disadvantage of this method is that the duplicate process 6 and the value of *Y* no longer contain all of the information about the original several process 5 lots that were entered.

### 2.2 Considering Time Delays

To construct a machine learning model, we created a dataset with the components of the raw materials and the process conditions, such as temperature and pressure, as *X* and the product properties as *Y*. A model *Y* = *f*(*X*) was constructed to express the relationship between *X* and *Y* using this dataset. However, the process under consideration was a dynamic process, in which the values of the process variables were continuously changing. Therefore, each variable was recorded with the value at the date it was measured, and there was no temporal linkage between *X* and *X*, and *X* and *Y*. We constructed the model to account for the imprecise time delays between *X* and *X* or *X* and *Y*.

### 2.3 GA‐based Process Variables and Dynamics Selection

The GAVDS method is a variable selection method based on a GA [31] that can simultaneously optimise the process variables and their time delays. Furthermore, the time‐delayed variables are continuously selected. For each process variable, the time‐delayed variables are prepared for each measurement time. When *k* process variables and time‐delayed variables are considered within a unit time *m*, the number of all variables is equal to *k* × (*m*+1). When the time width that is selected for a process variable indicates a region, the number of selected regions and the maximum value of the regions are set. In this study, the fitness of the GA was the determinant coefficient *r^2^* after five‐fold cross‐validation. The GAVDS program was written in Python using DEAP [32].

## 3 Results and Discussion

### 3.1 Model Construction

**FIGURE 2:**
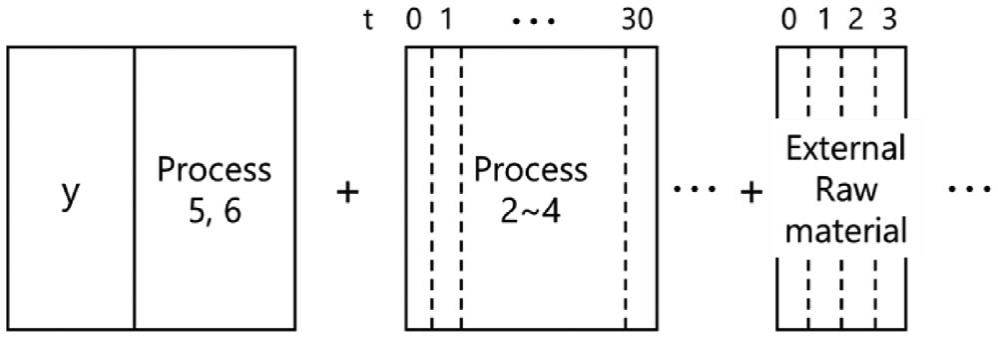
Created dataset for considering the time‐delayed process variables.

A schematic of the target processes is shown in Figure 1. From experience, we know that it took an uncertain number of days to go through each of the processes 1–4. There was no time delay for processes 5 and 6 for the products because they were operated in batch mode. We constructed a model that selectively performs GAVDS, which selects the process variables and time delays in processes 1–4, and GA, which selects only the variables in processes 5 and 6, as shown in Figure 2.

**FIGURE 3:**
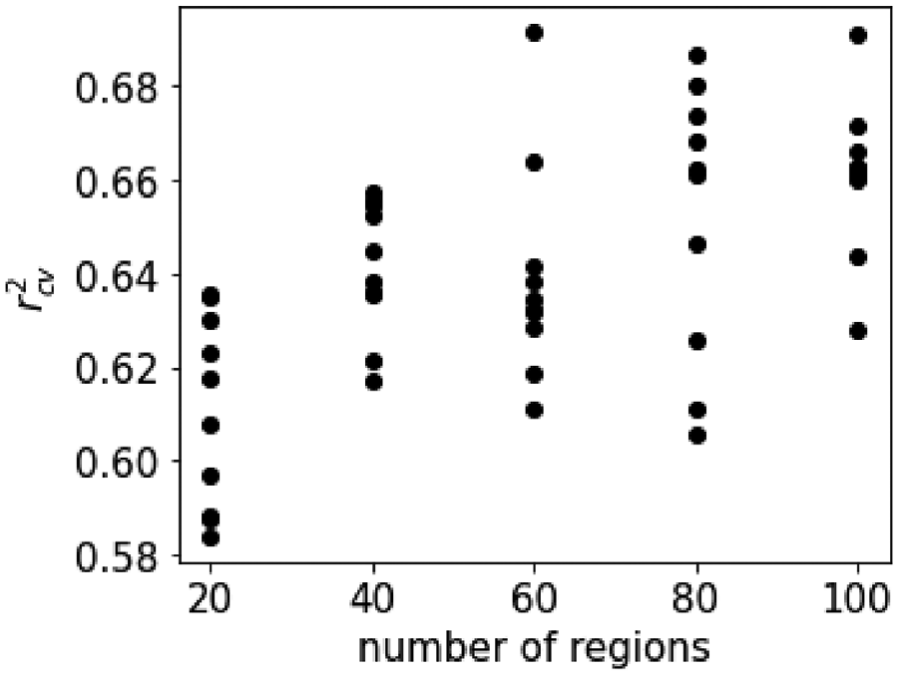
Comparison of the results of the number of regions (20–100) in the genetic algorithm‐based process variables and dynamics selection (GAVDS) model.

Based on experience, the time delays were estimated to be approximately 1 month for the entire process and approximately one week from process 4 to process 5. In addition, because an external raw material was loaded in succession in the process, we took the three most recent lots into account. We set a time delay of up to 30 days for processes 1 through 4, and up to the last three lots for the external raw material. With these settings, the dataset for GAVDS was created with 5418 variables that extend a process variable to one dimension by the amount of time delay you need to consider, as *X*. GAVDS was performed between these variables and thermophysical property A as *Y*. In this study, among the two thermophysical properties A and B, we focused on thermophysical property A because it better describes the quality of the needle coke. The number of generations was set to 100, and the number of populations was set to 100. The probability of crossover was set at 0.5 and the probability of mutation at 0.2. The regression method used was support vector regression with a Gaussian kernel. The *r^2^_cv_* value calculated by five‐fold cross‐validation [33] was used as an evaluation function of the chromosome. The maximum width of the regions was set to 20, and the number of regions to be compared was set to 20 increments from 20 to 100. The *r^2^_cv_* values of ten iterations of calculations with these settings are shown in Figure 3.

The best fit was obtained when the number of regions to be selected was 60 (Figure 3). We constructed a model to account for the time delays using 239 variables selected by the individual with the highest goodness of fit in the 60 regions to be selected.

### 3.2 Construction of the Regression Model

Models were constructed with and without time delays. The model without considering the time delays (no‐time‐delays model) used 569 variables as *X*, and the model considering time delays (considering‐time‐delays model) used 239 variables, which were selected as *X* for the GAVDS method. Thermophysical properties A and B were used as *Y* for regression analysis. We used 490 samples as training data and 76 samples as test data. The following regression methods were used:

- Ordinary least squares regression [34].
- Partial least squares regression [35].
- Ridge regression [36].
- Least absolute shrinkage and selection operator [37].
- Support vector regression with a Gaussian kernel [38].
- Decision tree [39].
- Random forest [40].
- Gradient boosting decision tree [41].
- Extreme gradient boosting [42].
- Light gradient boosting machine [42].
- Gaussian process regression [43].
- Gaussian mixture regression [43].

For each set of *x* values, the regression method that could construct the model with the highest predictive ability for the test data was used.

We used the mean absolute error (MAE) and the coefficient of determination ($r^{2}$) as an indicator to evaluate the estimation performance of the model:

(1) $$\mathrm{MAE}=\frac{\sum_{i=1}^{n}\left|y^{\left(i\right)}-y_{estimated}^{\left(i\right)}\right|}{n}\;$$

(2) $$\;r^{2}=1-\frac{\sum_{i=1}^{n}{\left(y^{\left(i\right)}-y_{estimated}^{\left(i\right)}\right)}^{2}}{\sum_{i=1}^{n}{\left(y^{\left(i\right)}-\hat{y}\right)}^{2}}\;$$

where *y^(i)^* is the actual value of the target variable for the ith sample, *y^(i)^_estimated_* is the predicted value of the target variable for the *i*th sample, $\hat{y}$ is the mean value of the target variable, and n is the number of samples. MAE is an indicator of the overall error magnitude, and the value is closer to zero for a smaller MAE value. *r^2^* represents the degree of variability of the objective variable that can be explained by the regression model. The closer the value is to one, the better the accuracy of the model can be evaluated.

**FIGURE 4:**
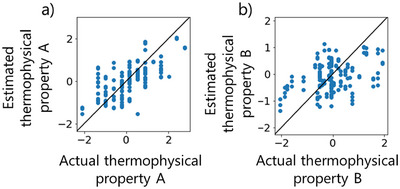
Actual *y* versus estimated *y* for the test data of thermophysical property (a) A (partial least squares regression) and (b) B (light gradient boosting machine) using the no‐time‐delays model.

**FIGURE 5:**
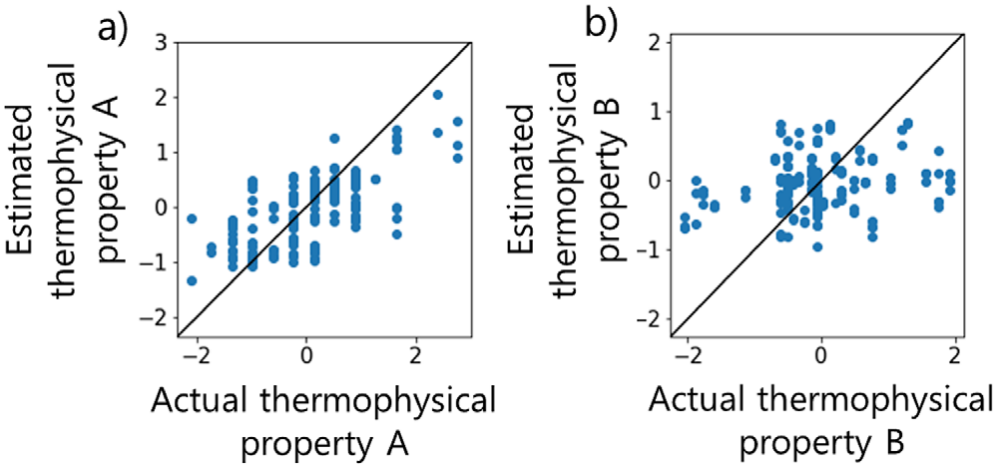
Actual *y* versus estimated *y* for the test data of thermophysical property (a) A (support vector regression with a Gaussian kernel) and (b) B (random forest) using the considering‐time‐delays model.

**FIGURE 6:**
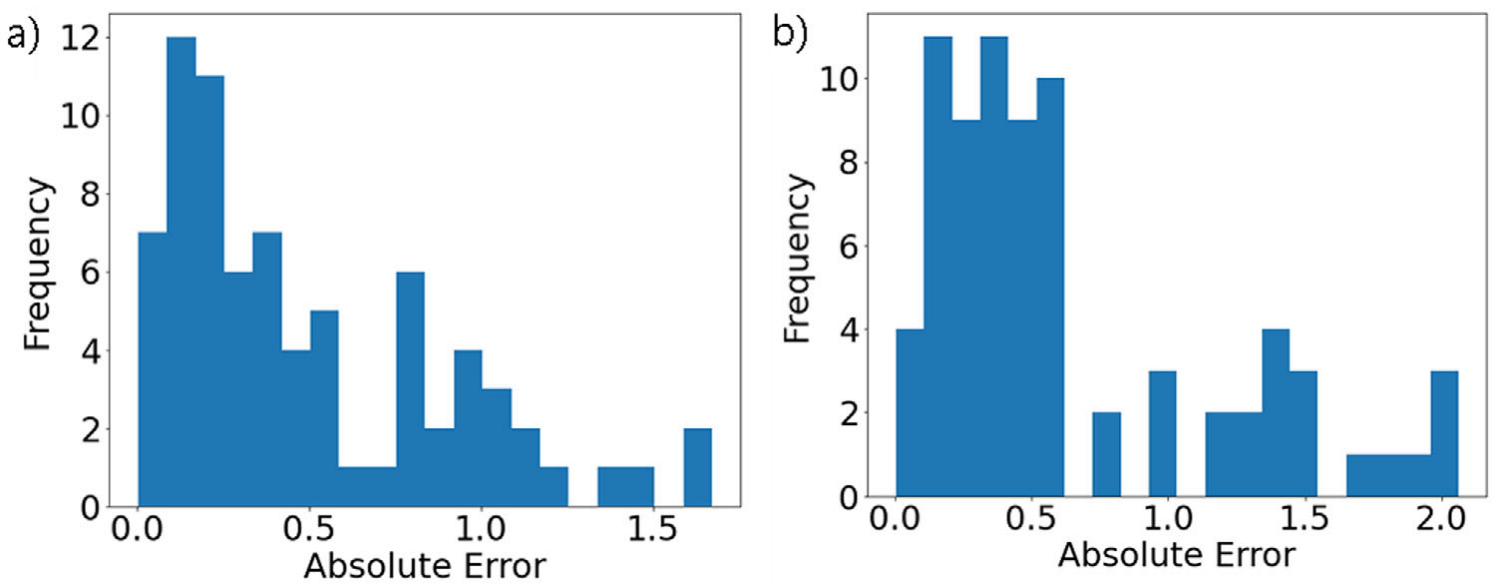
Mean absolute error (MAE) of the test data using the considering‐time‐delays model for thermophysical property (a) A and (b) B.

The smallest MAE values of all the regression methods in each model and the *r^2^* of that model are given in Table 2. Scatter plots of the values of the measured *Y* on the horizontal axis and the values of the estimated *Y* for each model on the vertical axis are shown in Figures 4 and 5. A histogram of the MAE between the estimated and measured *Y* values of the test data is shown in Figure 6.

**TABLE 2:** Results of the evaluation function mean absolute error (MAE) and *r^2^* for thermophysical properties A and B for the no‐time‐delays model and the considering‐time‐delays model.

Thermophysical property	Model	MAE	*r^2^*
A	No‐time delay	0.575	0.446
	Considering time delays	0.558	0.459
B	No‐time delay	0.696	0.063
	Considering time delays	0.686	0.053

The considering‐time‐delays models for both thermophysical properties A and B had smaller MAEs than the no‐time‐delays models. The maximum error of the GAVDS model was about 1.5 for thermophysical property A and about 2 for thermophysical property B. For the thermophysical property A of particular interest, the value of *r^2^* was larger for the considering‐time‐delays model than for the no‐time‐delays model. The small error indicates that the model with time delay has better predicted than the model without time delay. Furthermore, the scatter plots of measured and predicted values in Figures 4 and 5 show that the considering‐time‐delays model performed well because many of the plots are close to the diagonal line where measured values are high and predicted values are low.

### 3.3 Proposal of the Optimal Process Conditions

**FIGURE 7:**
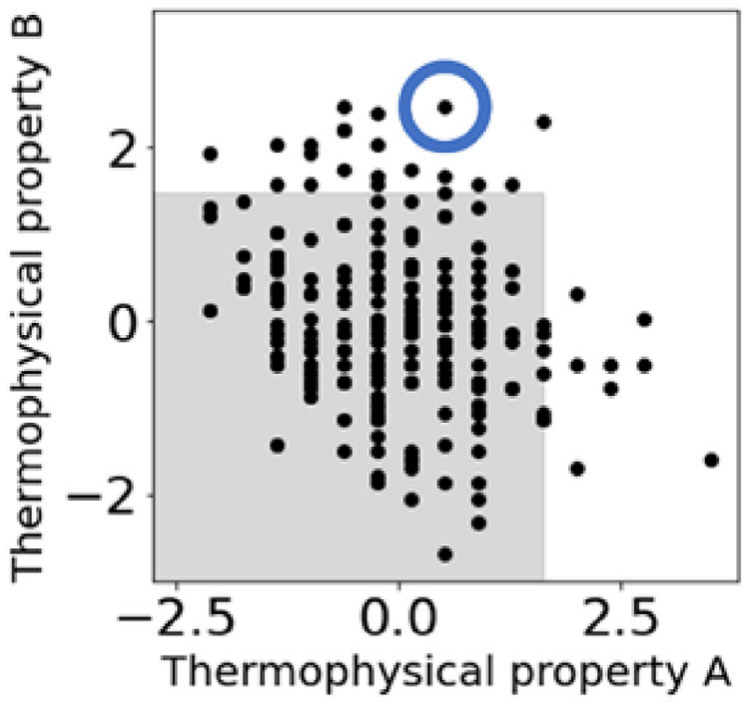
Scatter plots of the two thermophysical properties of the actual data. The grey area indicates the target area within the product specifications. The blue circle indicates a sample that had been considered to be outside the specifications.

Inverse analysis using the considering‐time‐delays model, which showed a small MAE, was performed. Among the *X* selected by GAVDS, the variable representing the components of the raw materials was fixed to the same value as a sample that had been considered to be outside the product specifications. The variable representing the process conditions was randomly generated with a range where the upper limit was set to the maximum value of past operation data, and the lower limit was set to the minimum value of past operation data. One million candidates were generated using these settings. The sample that was previously considered to be outside the product specifications is indicated by the blue circle in Figure 7. The considering‐time‐delays model was used to predict one million candidates and determine if they would fall within the target area (the grey area in Figure 7), within the product specifications. If the estimated *Y* value falls within the grey target area, it is possible to search for process conditions that will bring the product within the product specifications, whereas it would be outside the target area, and the product specifications if it were operated in the conventional way. Furthermore, if only the components of the raw materials are known, the appropriate process conditions can be proposed in advance.

**FIGURE 8:**
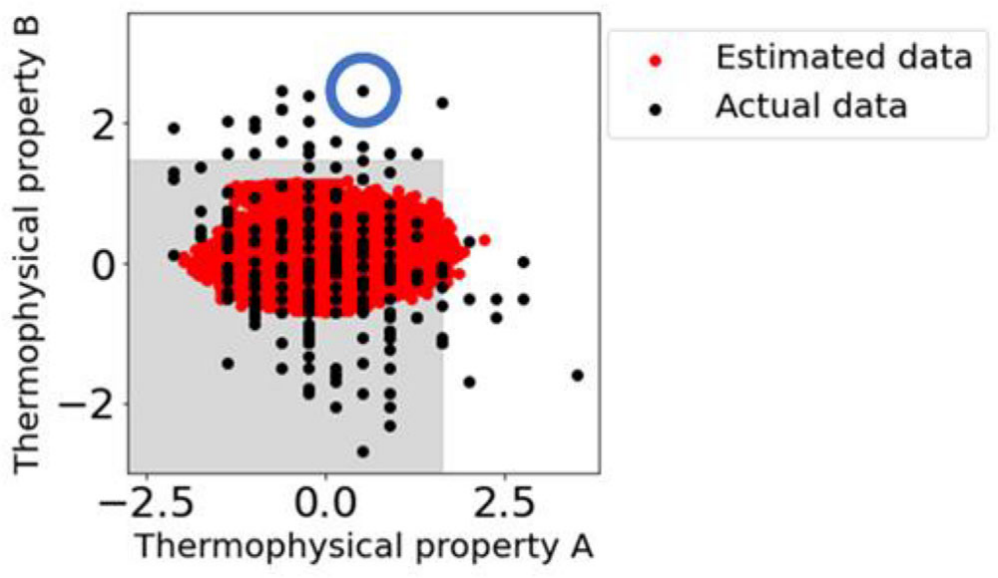
Scatter plots of the two thermophysical properties of the actual data and the estimated data of one million samples. The grey area indicates the target area within the product specifications. The blue circle indicates a sample that had been considered to be outside the specifications.

Approximately 930,000 of the one million generated samples fell within the grey target area (Figure 8). The thermophysical property B was estimated to be lower than the actual value when the value was high, and to be higher than the actual value when the value was low, as shown in Figure 5b. Therefore, when this model is used to predict the target sample, the value of the estimated *Y* is lower than the actual value. As mentioned in Section 3.2, the MAE of the one million generated samples is also expected to produce additional errors of up to 1.6 for thermophysical property A and 2.0 for thermophysical property B. For these reasons, we consider that the estimated value for the one million generated samples is close to zero, as shown in Figure 8.

## 4 Conclusions

When constructing a model for a dynamic process, it is necessary to consider the time delays. In this study, we constructed a model considering the time delays for processes with time delays in each process using the GAVDS method to express the relationship between the process and product properties. The prediction accuracy of the model considering the time delays was higher than that of the model without considering the time delays. We expect that the proposed method of optimising process variables and time delays by combining GA and GAVDS can be applied not only to needle coke production data, but also to other dynamic process data. However, if the process data are different, it is necessary to construct a model for each process data.

In the future, we will attempt to improve the accuracy of the prediction and to construct a model in which the MAE is small. We have three ways to improve the predictive accuracy of the model. The first one is a method of optimisation with two objective variables for time delay and process variable selection by GA. In this study, optimisation was performed so that the prediction accuracy of one objective variable would be high, but if the prediction accuracy of the two objective variables could be optimised simultaneously, it would be possible to capture the actual process dynamics more clearly. The second one is to incorporate knowledge from the manufacturing process into the model. By providing constraints based on knowledge at the time of manufacturing to our method, the model can reflect the relationship between actual process dynamics. The third one is to improve the prediction accuracy; the process variables need to be reduced as much as possible before using the GAVDS method. Because the GAVDS method simultaneously optimises the process variables and time delays, it generates as many *X* variables as the product of the number of variables and the maximum time delay. This can cause a combinatorial explosion of variables, and the solution can be a local solution. This can be seen from the large variation in the coefficient of determination shown in Figure 3. Therefore, pre‐selecting *X* variables before performing the GAVDS method is one way to improve the prediction accuracy.

## Conflicts of Interest

The authors declare no conflicts of interest.

## Data Availability

The data that supports the findings of this study are available in the Supporting Information of this article.
